# Bipolar electrochemical growth of conductive microwires for cancer spheroid integration: a step forward in conductive biological circuitry

**DOI:** 10.1038/s41598-024-71236-2

**Published:** 2024-09-09

**Authors:** Andie J. Robinson, Craig McBeth, Ruman Rahman, Richard J. M. Hague, Frankie J. Rawson

**Affiliations:** 1 Bioelectronics Laboratory, Regenerative Medicine and Cellular Therapies, Biodiscovery Institute, School of Pharmacy, University of Nottingham, Nottingham, NG7 2RD UK; 2 School of Health and Life Sciences, Teesside University, Middlesbrough, TS1 3BX UK; 3 Children’s Brain Tumour Research Centre (CBTRC), Biodiscovery Institute, School of Medicine, University of Nottingham, Nottingham, NG7 2RD UK; 4 Centre for Additive Manufacturing, Faculty of Engineering, University of Nottingham, Nottingham, NG8 1BB UK

**Keywords:** Wireless electrochemistry, Bipolar electrodes, Microwires, Cancer spheroids, Electrocatalysis, Biomedical engineering

## Abstract

The field of bioelectronics is developing exponentially. There is now a drive to interface electronics with biology for the development of new technologies to improve our understanding of electrical forces in biology. This builds on our recently published work in which we show wireless electrochemistry could be used to grow bioelectronic functional circuitry in 2D cell layers. To date our ability to merge electronics with in situ with biology is 3D limited. In this study, we aimed to further develop the wireless electrochemical approach for the self-assembly of microwires in situ with custom-designed and fabricated 3D cancer spheroids. Unlike traditional electrochemical methods that rely on direct electrical connections to induce currents, our technique utilises bipolar electrodes that operate independently of physical wired connections. These electrodes enable redox reactions through the application of an external electric field. Specifically, feeder electrodes connected to a power supply generate an electric field, while the bipolar electrodes, not physically connected to the feeder electrodes, facilitate the reduction of silver ions from the solution. This process occurs upon applying a voltage across the feeder electrodes, resulting in the formation of self-assembled microwires between the cancer spheroids.Thereby, creating interlinked bioelectronic circuitry with cancer spheroids. We demonstrate that a direct current was needed to stimulate the growth of conductive microwires in the presence of cell spheroids. Microwire growth was successful when using 50 V (0.5 kV/cm) of DC applied to a single spheroid of approximately 800 µm in diameter but could not be achieved with alternating currents. This represents the first proof of the concept of using wireless electrochemistry to grow conductive structures with 3D mammalian cell spheroids.

## Introduction

The field of bioelectronics is rapidly expanding. In particular, there is now a drive to merge electronics with biology, to input electrical stimuli, and modulate disease and is termed bioelectronic medicine. The limitations of modern bioelectronics go beyond size and performance, they are limited by the electronics themselves. In that, there is a mismatch between the material properties, the 3-dimensional (3D) characteristics and plasticity of biology which prevents the integration of electronics in biology and can lead to fouling through the body eliciting an immune response. Standard manufacturing processes for electronics produce hard/brittle structures, resulting in the lack of integration with 3D cell constructs. Hence, new 3D processes and materials are required to allow the production of nanoscale, flexible components that merge seamlessly with biological systems. As we learn more about bioelectricity and its importance in organ, tissue and cellular functions^1^, innovative bioelectronics are required to sense and actuate bioelectricity.

Many areas of the body are not accessible to large bioelectronic devices and implanted electrodes; therefore to fully exploit electricity as a therapeutic pathway we require novel means of approach to the design and manufacture of bioelectronics for real application. Liu et al. highlighted the need for bioelectronics to be redesigned: being 3D rather than planar, with micro/nanoscale feature sizes and comparable or softer mechanical properties than biological tissue^2^. Microfabrication methods, such as inkjet and screen printing, have been adapted using novel biomaterials to create soft, flexible electronics^3^. This has allowed skin-like electronics to be produced for novel applications such as tattoo sensors^4–6^, neural interfaces^7^, and in vivo electrochemical sensing of analytes such as cell metabolism^8^. This offers exciting possibilities for biosensing and wearable technology, however for, in vivo bioelectronic applications, soft electronics would still require implantation and provide only a 2D interface with biology.

Current micro and nanoscale electronics are dominantly manufactured using complex layering processes or 3D printing techniques, primarily extrusion printing or direct ink writing^9,10^. Whilst these processes can now provide flexible electronics to be wrapped onto biological material^11^, through the pre-assembling of the electronic component before their incorporation with biology, the interface between both systems is poor^12^, and only provides a 2D structure across a surface. Having the capability to self-assemble 3D electronics in situ would not only increase the information processing density of the electronics, but may also greatly improve the integration with biological tissue, and the efficacy of treatment.

One approach to addressing the challenges highlighted is by combining electronics with cells in vitro by to self-assembling conductive wire connects in situ in 3D. Self-assembly of 3D electronics has previously been shown using a range of materials^13–15^, and electrochemical techniques^16–24^. To merge such techniques with biology, a wireless approach is desirable, which is possible using bipolar electrochemistry^25,26^. Bipolar, or wireless, electrochemistry has been used previously to grow microwires (MWs) in the absence of biological systems^18,24^. We have previously shown wireless MW growth in the presence of cells (2D monolayers) which is possible using micro-bipolar electrodes (BPEs) stimulated under direct current (DC) ^27^. There have been no other reports of merging this method with 3D systems. Whilst previous studies have utilised electrochemistry to grow MWs to interface with individual cells^28^, none have yet to utilise this method with mammalian cells or to develop novel therapeutics/electroceuticals.

**Fig. 1:**
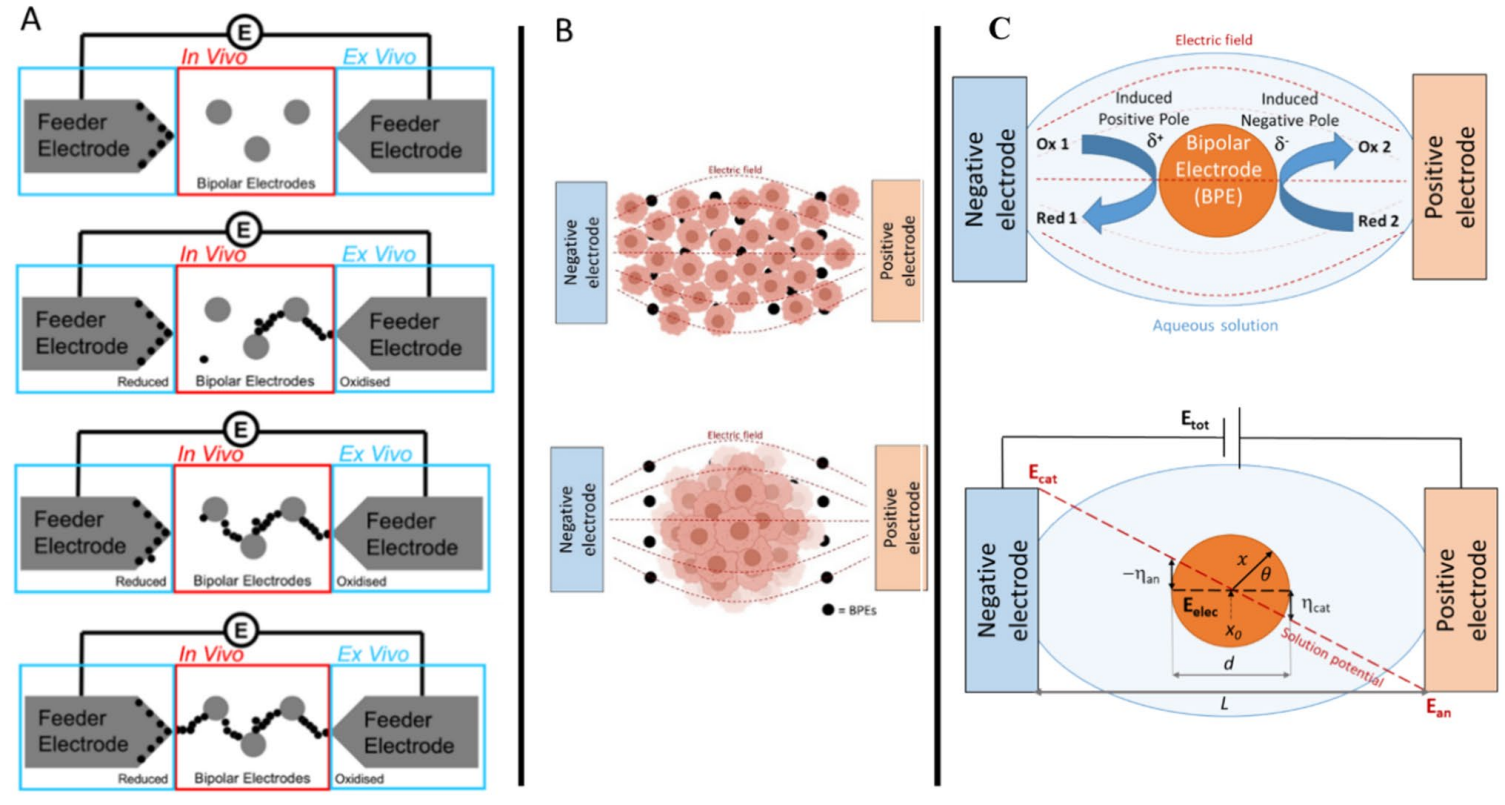
(**A**) Digramtic repsenation of wire growth mechanism using Ag as a simple proof of concept. For end application, BPEs model electrodes (red) whilst FEs (blue). (**B**) Wire growth set up with cells for 2D monolayers (Top), and 3D spheroids (Bottom). BPEs consisted of single ink drops of ~50 mm. Ibidi sticky-Slides were used to contain media in 10 x 10 mm areas around cells. Distance between FEs was equal to 1 mm. (**C**) Schematic of a bipolar electrode funtion in driving electrochemistry as a funtion of of potential. Noted in Eq. 2, the application of potential causes the printed Ag solid to donate an electron, and therefore form an ion in solution. This ion regains an electron and deposits to form wires (process shown in Fig. 1A) and continues to form wires.

Bipolar electrochemistry is a concept within electrochemistry, which forms the basis of the current work. Figure 1 shows a typical set up of an open bipolar electrochemical cell. A an electrochemical setup includes feeder electrodes (FEs) (positive and negative), which are attached to a power source; one or many bipolar electrodes (BPEs), which are not attached to a power source; and an aqueous electrolyte solution. FEs are used to apply a uniform electric field across the electrolyte solution. This electric field (E_tot_) will polarise conductive objects within it, and hence the bipolar electrode will then possess two poles (d^+^ and d^−^) that can act as an anode and cathode in electrochemical reactions^25^. Due to the bipolar electrode being conductive, it’s potential (E_elec_) is in equilibrium across its surface. However, the presence of the electric field means the interfacial potential difference between the BPE and the solution varies along the BPEs length. It is these varying overpotentials (η_an_ and η_cat_) that drive electrochemical reactions at the poles of the BPE^25,26^. The poles of the BPE are orientated in the opposite polarity to the FEs^5^, with the highest overpotentials being generated at the extremities of the BPE. The location on the BPE at the boundary between the two poles has zero overpotential with respect to the solution (*x*_0_). Although this is in the centre of the BPE in Fig. 1, its actual location depends on the faradaic processes taking place at the poles of the BPE. Our premise in this work was to use silver electrochemistry to grow microwires at bipolar electrodes (Eq. 1) to interface with cancer spheroids.

Many design parameters are responsible for controlling bipolar electrochemical processes. These include the potential applied to the system (E_tot_), the distance between the FEs (L), and the length of the BPEs (d). The proportion of E_tot_ that is dropped over the BPE can be described as E_elec_. This can be calculated using Eq. 1. It is important to note that this assumes a linear electric field: meaning the geometry of feeder electrodes is the same and that the BPE is not significantly affecting the electric field. To note though the Rawson group have shown at the nanoscale these assumptions break down and much is still to be understood about wireless electrochemistry from a theoretical point of view^27,29,30^.

1 $$\Delta E_{elec} = E_{tot} (d/L) = \eta_{an} - \eta_{cat}$$

In order for an electrochemical reaction to take place, this potential drop E_elec_ must exceed the difference between standard redox potentials of the anodic and cathodic reactions. An example present in this thesis is the REDOX of Ag to generate Ag microwires (AgMWs). As seen in Eqs. 2 and 3, in order to drive this reaction E_elec_ must exceed ~ 1.63 V.

2 $$Ag(s) \leftrightarrow Ag^{{ + {\text{aq}}}} + e^{ - } \leftrightarrow Ag^{ + } (s) + e^{ - } + 0.7996$$

3 $$2H_{2} O + 2e^{ - } \leftrightarrow H_{2} (g) + 2OH - 2H_{2} O + 2e^{ - } \leftrightarrow H_{2} (g) + 2OH^{ - } - 0.8277$$

Herein we build on our previous work in which we demonstrated growth of functional bioelectronics in 2D cell layers and begin to address these challenges by improving our understanding of the optimal inputs for MW growth, understanding the effect of bipolar electrochemical system (BES) design (i.e., geometry, orientation, and size of BPEs) on MW growth and optimising MW growth using AC current. This allows for proof of concept for the merging of in-situ grown bioelectronics with 3D cell cultures.

## Methods

### Materials

Materials were purchased from Sigma-Aldrich/Merck (UK) and utilised as received unless stated otherwise.

### Electrode Consideration

To produce a BES that enabled the growth of nano-electronics in combination with mamilian cells and propigated with 2D feeder electrodes (FEs) and BPEs were designed. Silver nanoparticle (AgNP) ink has been widely characterised for research purposes^31^, and allows for reproducible^32,33^. Silver electrochemistry allows for simple reduction and oxidation in deionised water (diH_2_O), with the application of potential as the driver (Fig. 1). Previous studies have shown Ag to be suitable for wirelessly growing electronics with cells without perturbations to cell viability^27^. Exemplar electrode patterns are shown in Fig. 1, forming simple BESs.

### Electrode manufacture

Inkjet printing was utilised to produce feeder electrodes, and a piezoelectric drop on demand Dimatix Materials Printer (Model DMP-2800, FUJIFILM, Dimatix, Inc. Santa Clara, CA) was used. Glass substrates were used, either in the form of glass slides (76 × 26 mm, Cole-Parmer) or glass coverslips (Ø 22 mm, Agar Scientific). Inks were filtered using HPLC Nylon 5 µm syringe filters (WZ-32816-14, Cole-Parmer), before being injected into print head reservoirs. Glass substrates were cleaned using Piranha solution (1:3 ratio of hydrogen peroxide (30%) to sulphuric acid (98%), H_2_O_2_:H_2_SO_4_) prior to printing.

\To allow for optimal wetting of the inks and stability in aqueous solutions, a polymer was first printed to aid adhesion^27^. A tri(proplylene) glycol diacrylate (TPGDA) ink was used, which had been modified to polymerise under UV light^34^. For this purpose, a UV light was mounted onto the print head (365 nm and 6000 mJ cm^−2^, Printed Electronics Ltd.). To prevent polymerisation inside the cartridge, the reservoir was sealed with duct tape. Ag nanoparticle (AgNP) ink (Advanced Nano Products Ltd.) with a solid content of ~ 30% was used for printing conductive tracks. An in-house heated substrate was used to heat the ink to 100 °C during printing; full sintering was performed using a 200 °C oven.

**Fig. 2:**
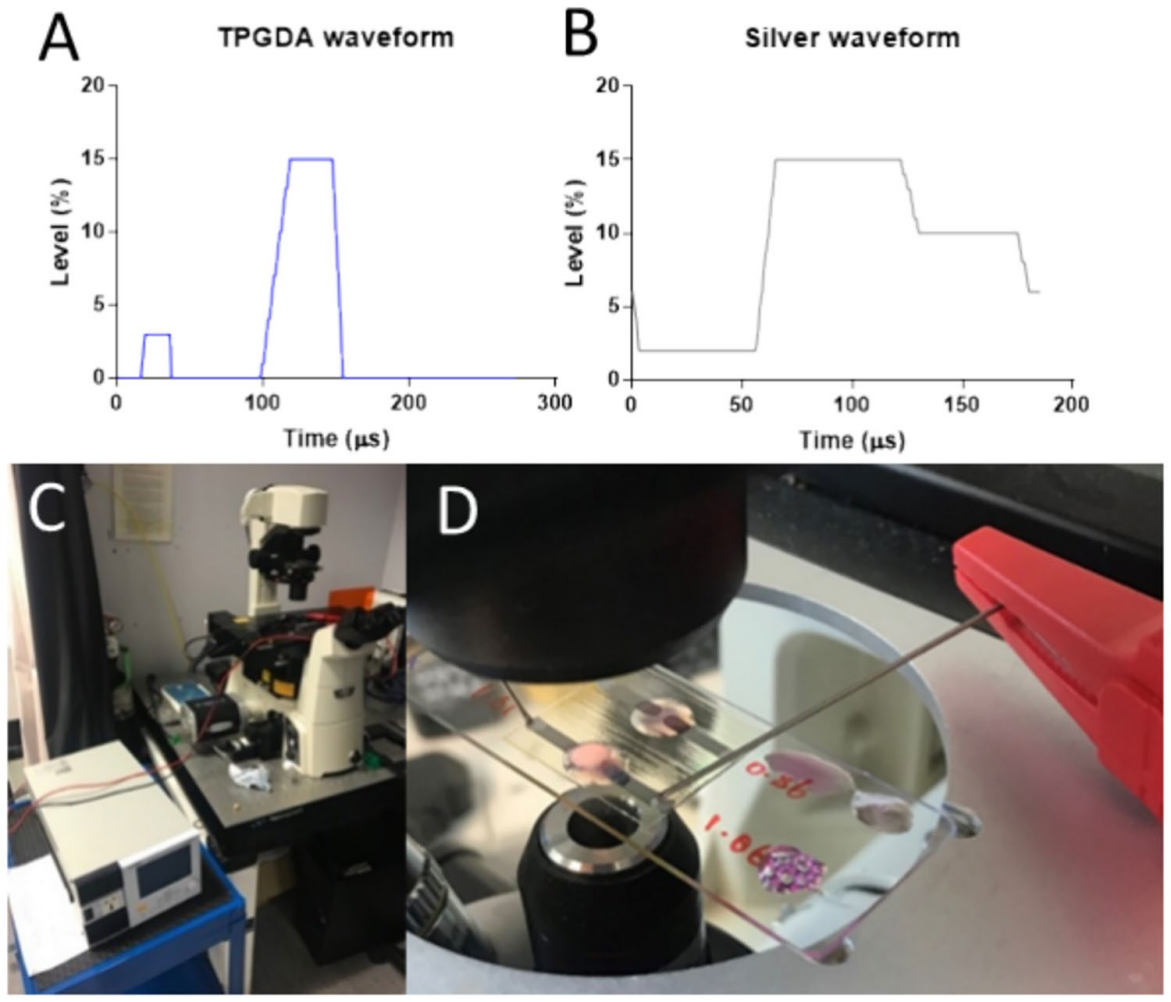
Jetting waveforms for printing (**A**) TPGDA and (**B**) Silver using a piezoelectric drop-on-demand printer. (**C**, **D**) Wire growth set up, using an optical microscope, high voltage stimulator (HVS), Ag paint contacts and needle electrodes.

Five layers of TPGDA were printed to allow for a level surface. Three layers of AgNP ink were printed to improve conductivity, yet still, allow for a short print time. Waveforms used for each ink were optimised and can be seen in Fig. 2 A + B. The print resolution was 846.67 drops per inch, corresponding to a drop spacing of 30 μm. The firing voltage varied over a range of 24−30 V.

### Electrochemical wire growth

Ag wire growth was performed in diH_2_O and applying varying electrical inputs with a high voltage stimulator (HVS–EC100SA, NF Instruments). As shown in Fig. 2 C + D, wire growth was imaged in real-time using a Nikon ECLIPSE TE300 fluorescent microscope equipped with a QImaging optiMOS Scientific CMOS camera. To improve electrical contact between the power source (HVS) and printed electrodes, silver paint (186–3600, RS components) was applied to the 3D printed samples and disposable 20 gauge steel needles (304,827, BD) were used to attach samples to HVS.

Wires were characterised using a wireless TRMS multimeter (EX542, Extech Instruments) to measure resistance and imageJ used to measure wire diameter from microscopy images (n = 10, randomly chosen).

### Electrochemical impedance spectroscopy

All impedance measurements were carried out using an Autolab potentiostat (Metrohm) with an applied AC amplitude of 0.01 V_RMS_. A two-electrode system was used, with a working electrode and reference/counter electrodes combined. Impedance was measured between 10 and 10,000 Hz, with a current limit set at 1 mA.

### Cell culture

Human glioblastoma (grade 4 brain tumour) cells (U251) purcahsed form ATCC were cultured in T-75 flasks in Dulbecco’s modified Eagles high-glucose medium (DMEM) (supplemented with 10% FBS, herein referred to complete DMEM (C-DMEM)) at 37 °C and 5% CO_2_ and passaged at 70–80% confluency. To culture monolayers onto 3D printed electrodes, modified adhesive wells (80,828, Ibidi) were utilised and adhered to electrodes, providing a 1cm^2^ cell growth area. Electrodes were plasma cleaned and silanized to increase the adhesion of printed structures and prevent detachment in an aqueous solution. Glass slides were immersed in ethanol for 10 min before O_2_ plasma cleaning. Glass slides were placed into a plasma chamber between two copper band electrodes connected to a power source (Coaxial Power Ltd.). The chamber was evacuated, and needle valves (BOC Edwards) were used to control the oxygen pressure. The pressure was monitored with a Pirani gauge (Kurt J. Lesker Ltd.) and a glow-discharged plasma was initiated when the pressure was stabilised to 9 × 10^–2^ Mbar (50W for 3 min). Salinisation was performed using 1% 3-(Trimethoxysilyl) propyl methacrylate (TMSPMA) in toluene for 1 h, followed by washing with dH_2_O. Cells were seeded at ~ 5 × 10^5^ cells per well in 300µL of culture media. Immediately before wire growth, media and sticky slides were removed, samples were washed with PBS, and substituted with a droplet of diH_2_O.

3D spheroids were cultured using a Rotary Cell Culture System (RCCS, Synthecon). Cells were incubated in disposable 10 ml culture vessels for 3–4 days and rotated at varying speeds to ensure spheroids were in a constant state of free-fall. Spheroids were characterised using light microscopy (Diaphot 300, Nikon). To initiate wire growth, cultured spheroids were washed in PBS and placed between 3D printed electrodes, where diH_2_O was added, covering the spheroid.

## Results and discussion

### Satellite BPEs

**Fig. 3:**
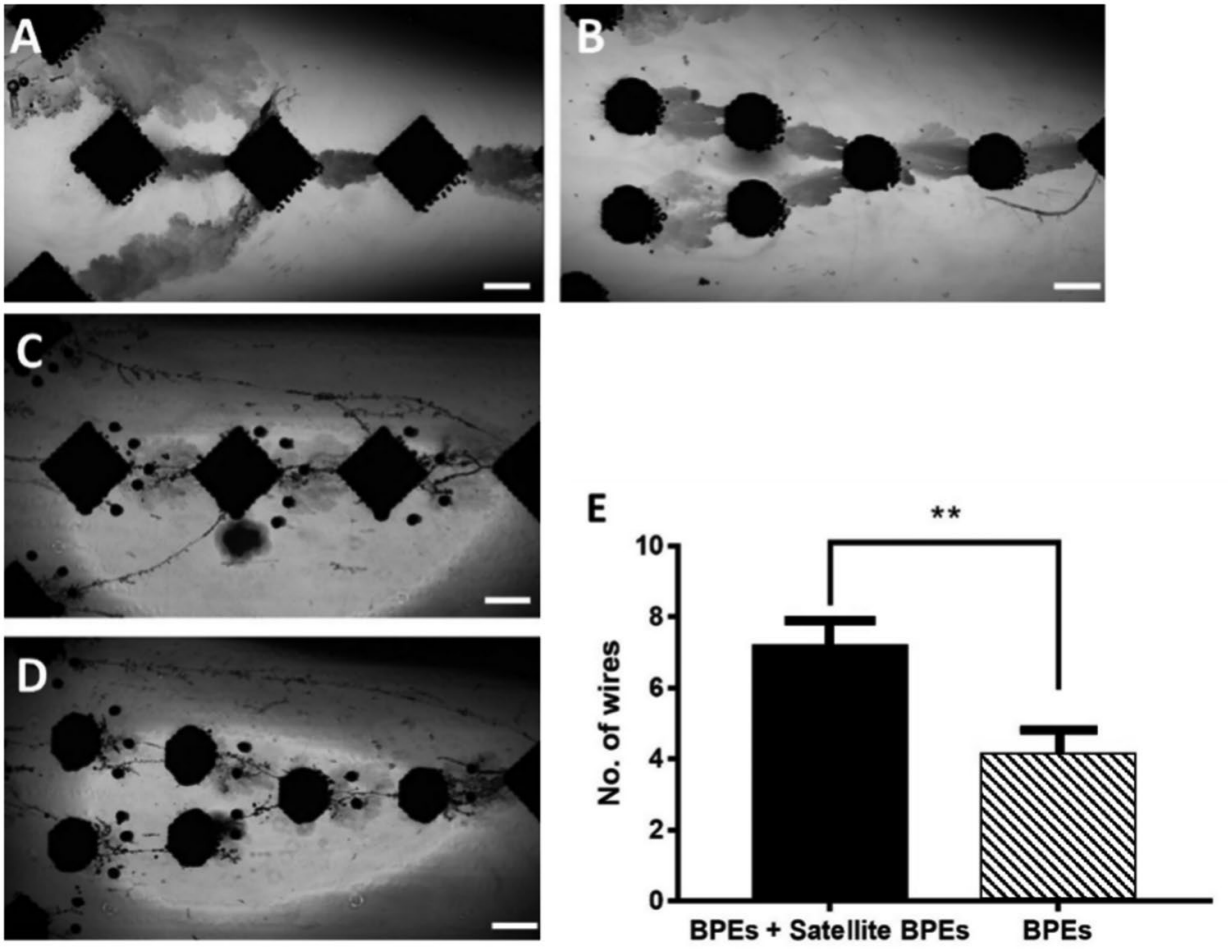
Comparison of number of MWs grown using samples with and without satellite BPEs. Multiple geometries were used in pairs, without satellite BPEs (**A**, **B**) and with (**C**, **D**) satellite BPEs (scale bar = 250 μm). (**E**) Mean + /− SEM of number of MWs grown demonstrates a significant increase in MW growth in the presence of BPEs (*P* < 0.05) (n = 20).

In previous work^27^, we have shown diamond BPEs out-perform round or triangular BPEs with regards to linkage formation. An electrode point was mathematically modelled by two spherical electrodes connected by a wire^35^. Based upon this we hypothesise MW growth will be enhanced by incorporating smaller BPEs close to the larger BPEs which will lower cellular resistance providing directional cues for the pattering of microwire growth. To evaluate this, silver MW growth with smaller BPEs incorporated were investigated (Fig. 3). Single droplet BPEs were explored (30–50 mm), herein referred to as satellites. As demonstrated in Fig. 3A–D, satellite BPEs were printed around larger BPEs, and comparisons to larger BPEs were evaluated. This demonstrated that incorporation of satellite BPEs significantly increased the number of wires grown against BPEs alone (4.1 ± 0.73 to 7.2 ± 0.69, respectively (n = 20) as shown in Fig. 3E). This finding will impact the design of systems for the wireless growth of MWs and suggests that multiple-sized electrodes should be incorporated. We suggest this occurs due to a drop in cell resistance.

As per Eqs. 4 and 5, it is hypothesised this observed behaviour is due to smaller volumes possessing a higher charge density resulting in an enhanced electric field. Hence, electrodes with a pointed edge, rather than a smooth/rounded edge, are predicted to possess improved MW growth due to an enhanced electric field at their points. We have recently substantiated this with a nano-bipolar electrode which results in the need to apply smaller currents than predicted^29^. Whilst smaller BPEs concentrate charge to act as nucleation sites for MW growth to initiate, larger scale BPEs are still required to lower the overall impedance of the system and allow for lower potentials to be used^36^.

4 $$\nabla \cdot E= \frac{\rho }{{\epsilon }_{0}}$$

where $\nabla =$ Divergence, E = electric field, $\rho =$ charge density and ${\epsilon }_{0}$ = permittivity

5 $${\uprho } = \frac{{\text{q}}}{{\text{V}}} $$

where q = charge and V = volume of electrode.

### AC MW growth

We next sought to establish and optimised the MW growth procedure for later cellular studies. We have previously demonstrated that AC stimulation may improve cell viability in a bipolar electrode system^27^. With the introduction of a frequency component, we hypothesised that this could allow for alternative electrical inputs that are less harmful to cells. Tissue damage is often a result of water electrolysis and noxious by-product from chemical reactions; utilising AC stimulation may reduce this due to less charge building up. AC stimulation has been used previously to grow MWs, although these also required the use of harmful agents that would not be suitable for use with biology^19,23^. Small single drop BPEs (30–50 µm) were incorporated between two FEs, replicating previous DC studies^27^, using varying potential ranges (1–100 V) and frequency ranges (1 Hz–1 MHz), and stimulation was applied to attempt to elicit MW growth.

**Fig. 4:**
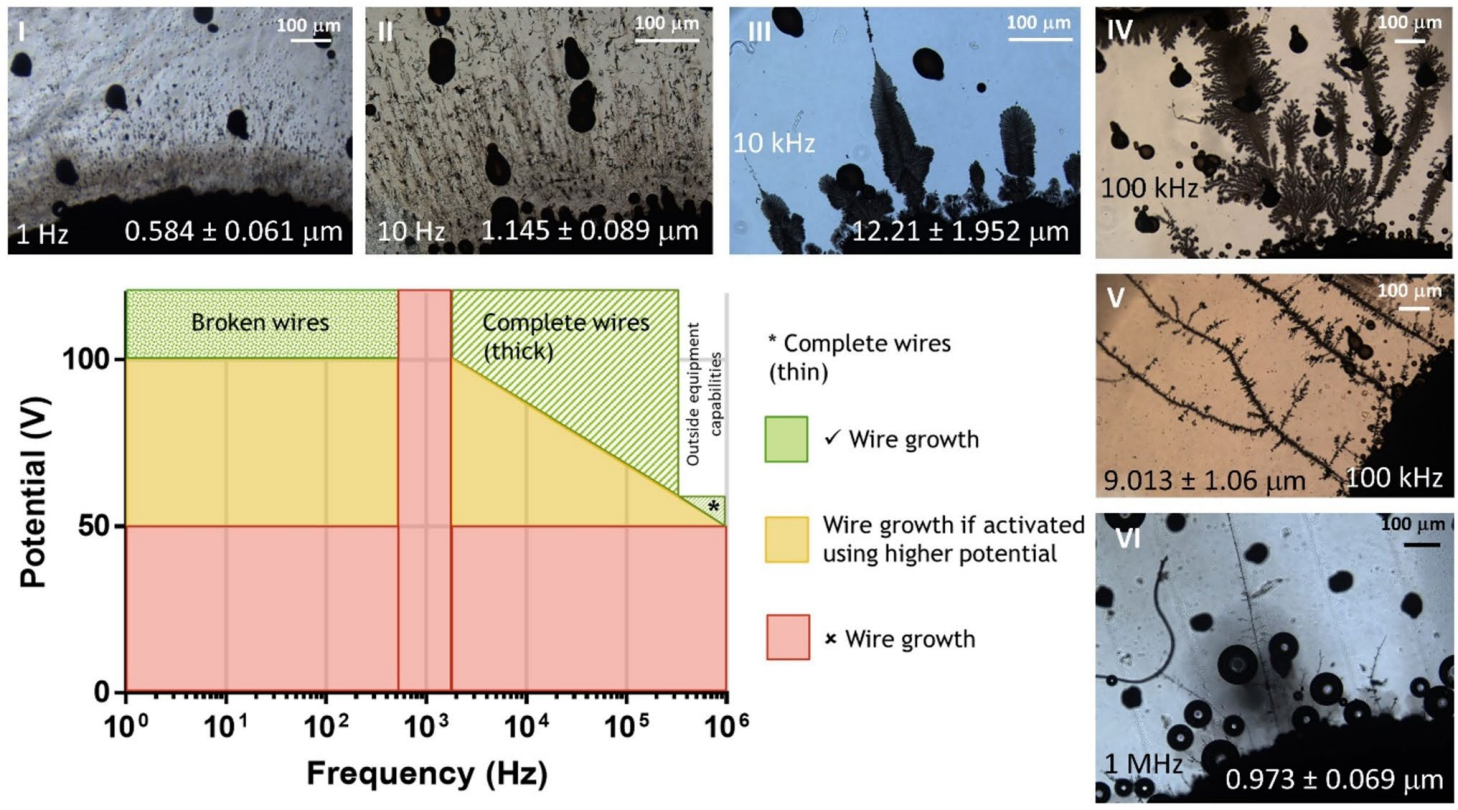
AC MW growth attempted at varying frequencies and potentials. Characteristic wires for varying frequencies at 100 V (or 50 V at 1 MHz) can be seen in images I–VI.

As shown in Fig. 4, MW growth was not possible at 10 V for any frequency, similarly, MW growth was not possible at 1 kHz. The exact reason for this is unclear and requires further investigation. Figure 4 insets I–VI show varying frequencies producing observably different MWs. At frequencies > 100 Hz, broken MWs form that are 0.5–1 mm; these MWs form when using 100 V, or by applying 100 V for 2 s before lowering to 50 V. Higher Frequencies (10 kHz) initially produce MWs with a wider diameter, with large amounts of amorphous growth. Wire thickness gradually decreases becoming less amorphous to ~ 1 mm at 1 MHz. This proportionality of decreasing diameter at higher frequencies is comparable to AC MW growth using other materials^22,23^, although wire thickness is magnitudes larger. Ten and 100 kHz allowed for MWs to be grown at 50 V when applying 2 s of 100 V to initiate MW growth. Furthermore, 1 MHz induced growing MWs at 50 V, 100 kHz growing MWs between 60 and 70 V, and 10 kHz growing MWs between 80 and 90 V.

One of the critical advancements ultimately needed to facilitate in vivo MW growth is a method to non-invasively determine if growth has occurred. Visualising wire growth within 3D cell cultures is challenging, and these difficulties will likely be amplified in tissue samples and animal models. Therefore, a wireless monitoring technique for wire growth would be highly valuable.

**Fig. 5:**
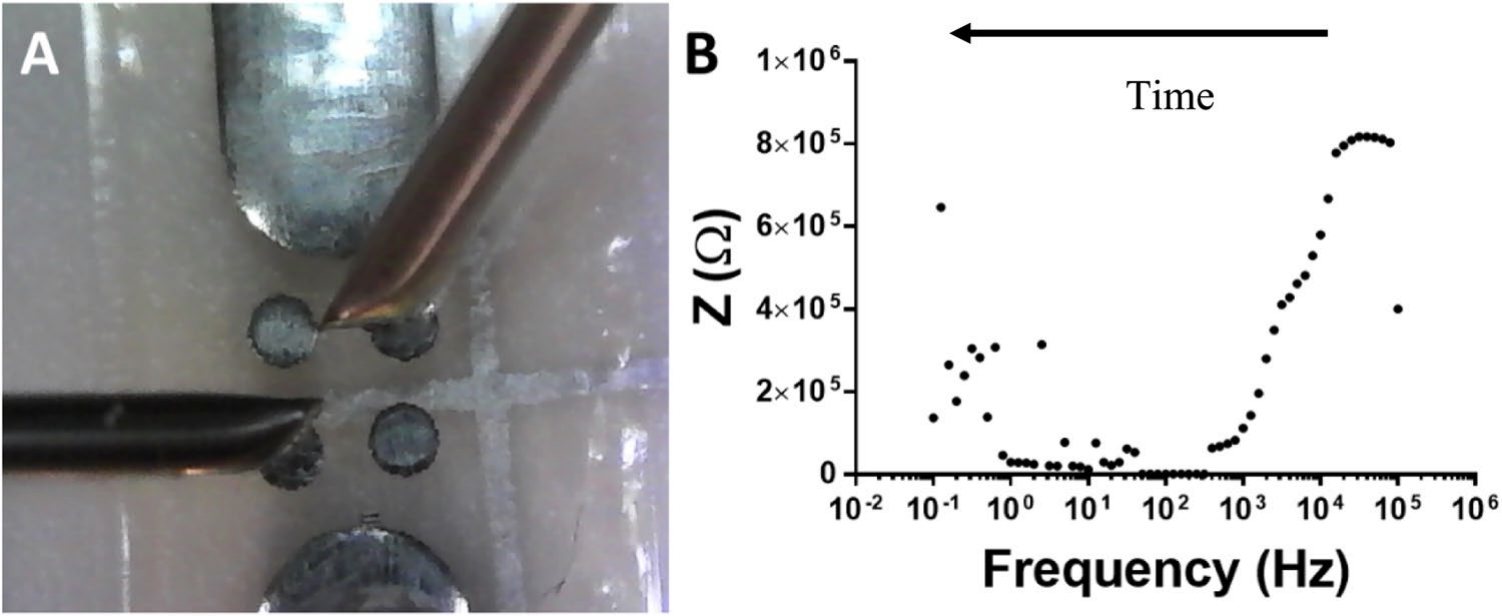
Impedance measurements during wire growth, Set up (**A**) and example bode plot in galvanostatic mode (**B**).

**Fig. 6:**
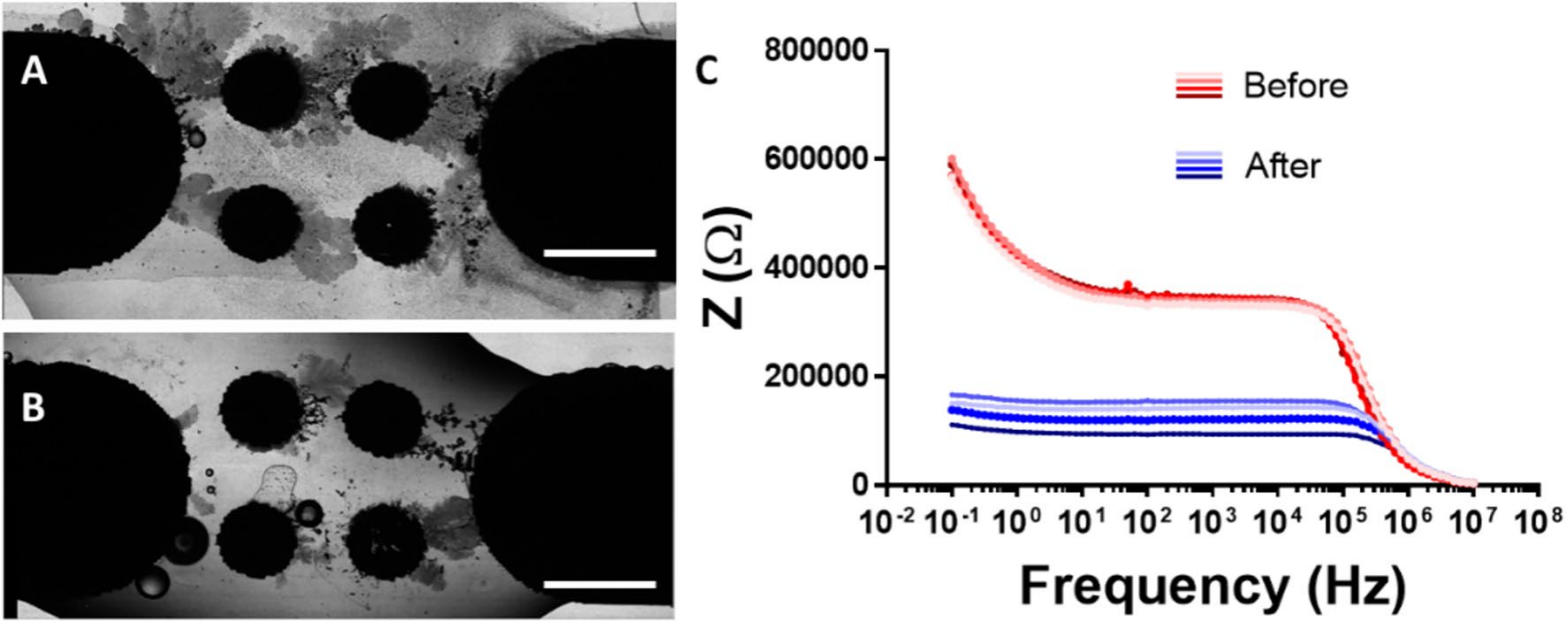
Example wire growth samples can be seen in A and B, scale bars show 100 μm. Impedance measurements before and after wire growth (C).

Electrochemical Impedance Spectroscopy (EIS) has demonstrated its effectiveness in probing nano-bipolar electrodes (nano-BPEs), making it a promising candidate for this application. Initial studies, conducted in the absence of cells, involved measuring the impedance BES before and after wire growth. A potentiostat was connected to two BPEs (illustrated in Fig. 5A), and EIS measurements were performed in galvanostatic mode during MW growth, resulting in Bode plots (Fig. 5B). EIS was used to assess the overall BES impedance before and after MW growth. Results (Fig. 6) consistently showed that MW growth led to a reduction in the BES's impedance.

The Bode plot depicted in Fig. 5B captures the impedance dynamics under galvanostatic conditions, where the current remains constant, across varying frequencies during the growth of microwires. Unlike cyclic voltammetry, where time is a direct variable, this plot represents impedance as a function of frequency. The directional indication on the graph refers to the progression of the impedance scan over time. Although the plot initially shows an higher impedance with frequency, the overall trend indicates a decrease in impedance as the microwire matrix develops and the system's conductivity enhances leading to lower impedance at lower frequencies when analysed.

Contrastingly, Fig. 6B presents a comparative view of impedance measurements taken before and after microwire growth. This figure demonstrates a decrease in impedance as a result of the formation of conductive paths within the system, reflecting a static comparison rather than the dynamic changes during microwire formation observed in Fig. 5B. This distinction is crucial for understanding the dynamic versus static nature of impedance changes associated with microwire growth and system conductance.

### 3D cell culture MW growth proof of concept

**Fig. 7:**
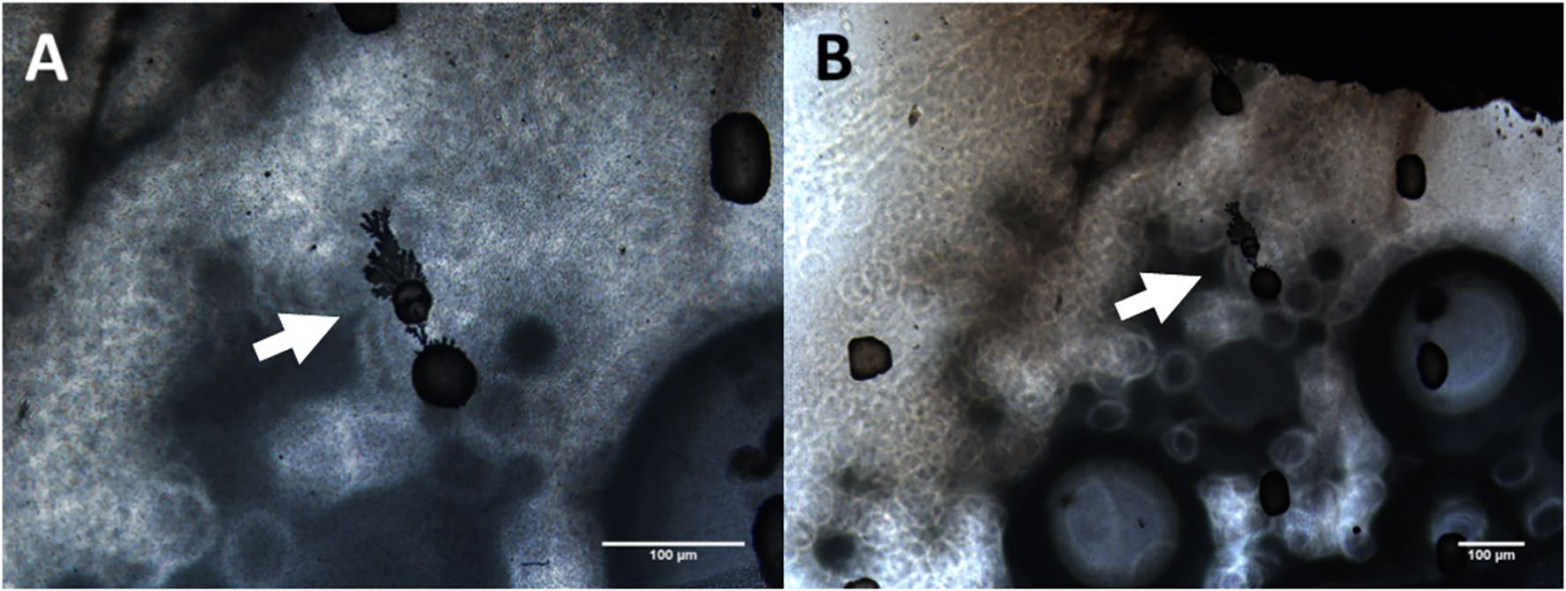
(**A**, **B**) Optical images of MW growth using 50 V at 1 MHz in the presence of 2D monolayers. Arrows highlight MWs. Scale bars represent 100 μm.

Whilst others have applied 3D Kirigami electronics for interfacing with 3-dimonsional culture. These can still not be assembled in situ^37^. To utilise this method for wirelessly growing in-situ bioelectronics, it is necessary to control microwire growth in the presence of biology. As we have demonstrated, microwire growth is possible in a 2D space, however, the next stage requires microwire growth in a 3D cell culture model. Glioblastoma cells (U251) were chosen for future applications of in vivo grown bioelectronics in neural-based therapeutics. Spheroids were attached to BPEs between two FEs (1 mm apart) using a coating of poly-D-lysine. Average spheroid diameter ranged from $\sim$ 100 to 400 μm, hence 2–3 spheroids were attached between FEs that were 1 mm apart. AC electrical stimulation was then used to attempt wire growth in the presence of the spheroids where; identical electrical inputs as above were used. As shown in Fig. 7, MW growth was possible at 50 V (0.5 kV/cm), 1 MHz; however, these were limited in length (50 mm) and required high potential, resulting in cell clumping. It was expected that wire growth was not possible at other inputs due to spheroids introducing high impedance to the BES.

**Fig. 8:**
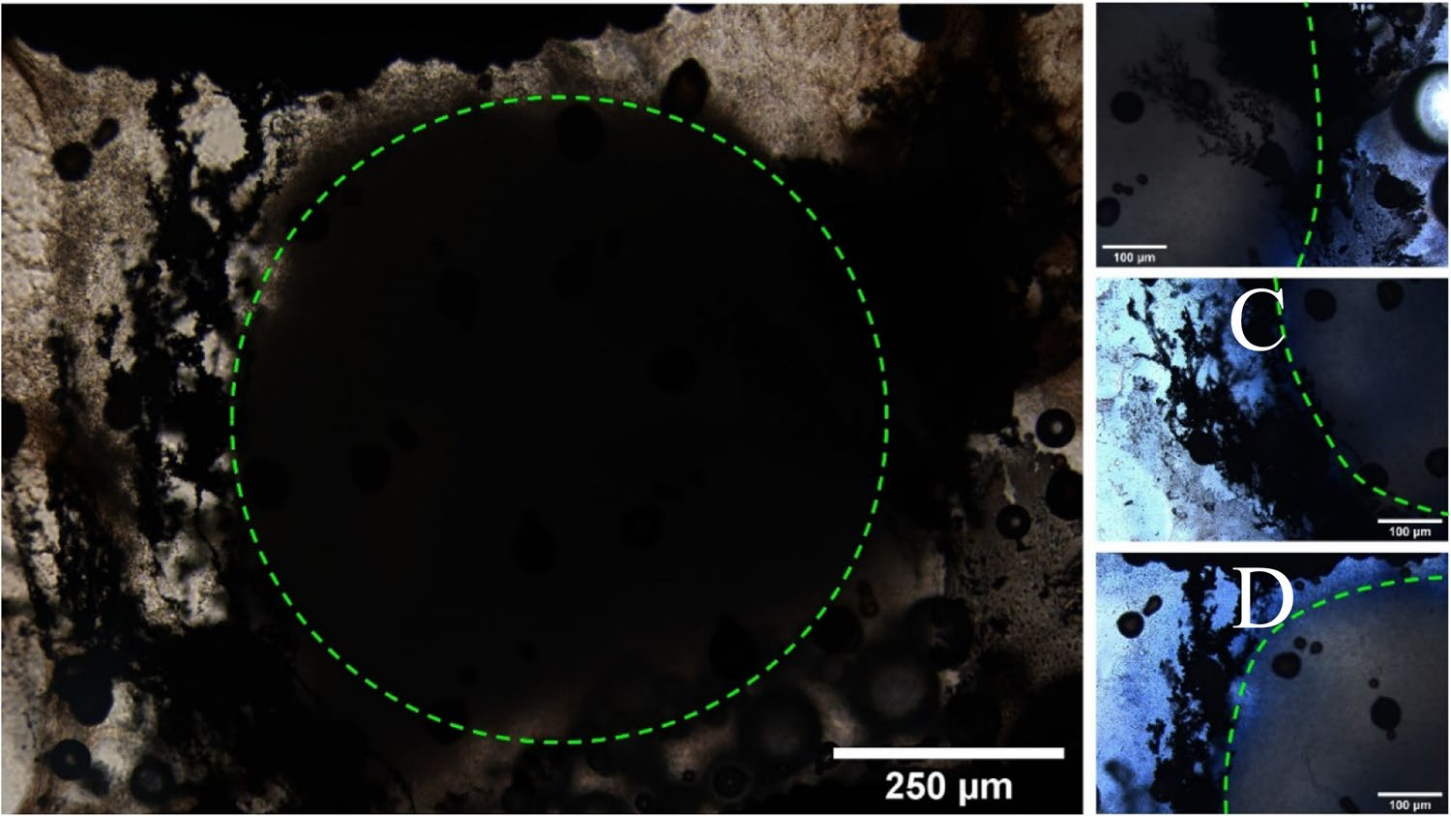
Optical images of Ag MW growth in the presence of spheroids. Green dotted outlined shows the main bulk of the spheroid. (**A**) whole image of MW growth around a spheroid, (**B**–**D**) Higher magnification of spheroid perimeter with Trypan blue stain to demonstrate MW growth.

As AC stimulation was not capable of growing MWs in the presence of cell spheroids, DC stimulation was pursued. As shown in Fig. 8, MW growth was successful when using 50 V (0.5 kV/cm) of DC applied to a single spheroid of approximately 800 µm in diameter. Wires were grown at 50 V using pulsed power supply in 10 pulses of 300 ms with a gap between pulses of 200 ms for until wires had been observed to have grown for approximately 60 s. It can be observed that MWs successfully grew around and underneath spheroids with a diameter of 7.89 ± 0.6 µm (n = 50), which is similar to electrical inputs without cells (Fig. 6). This suggests that the wire structure is similar to studies formed from AgNPs alone.

To approximate the effect of MW growth on cell viability, trypan blue was applied to spheroids (Fig. 8B–D). This suggested that a small population of cells had broken away from the surface of the spheroid and were not viable; nevertheless the main cell population’s membranes appeared still intact.

## Conclusions

This is the first 3D proof of concept using wireless electrochemistry to grow conductive structures with cell spheroids and hence is an exciting addition to the existing literature. Whislt AC stimulation was not capable of growing MWs in the presence of cell spheroids, MW growth was successful when using 50 V (0.5 kV/cm) of DC applied to a single spheroid of approximately 800 µm in diameter. This shows future promise that we may utilise the above and similar electrochemical methods to grow conductive structures in vivo*.* As 3D cell spheroids are often used to model tumours, this suggests we may be able to grow MWs around tumours for the study of manipulating cancer preocesses.

## Data Availability

The datasets used and/or analysed during the current study available from the corresponding author on reasonable request.
